# Plexiform Schwannoma of Lumbar Region

**Published:** 2015-05-01

**Authors:** Asmita Parihar, Sarika Verma, Tarun Suri, Anil Agarwal, Kalpana Bansal, Ruchika Gupta

**Affiliations:** 1 Department of Pathology, Chacha Nehru Bal Chikitsalaya, Geeta Colony, Delhi-110031, India; 2 Department of Orthopaedics, Chacha Nehru Bal Chikitsalaya, Geeta Colony, Delhi-110031, India; 3 Department of Radiodiagnosis, Chacha Nehru Bal Chikitsalaya, Geeta Colony, Delhi-110031, India

**Keywords:** Schwannoma, Plexiform, Soft tissue, Lumbar

## Abstract

Plexiform schwannoma is an unusual peripheral nerve sheath tumor. It can mimic plexiform neurofibroma. A five-year-old girl presented with painful swelling in left lumbar region. Radiologic investigations showed a multinodular tumor in the subcutaneous plane of lumbosacral region. A complete excision and histopathologic examination revealed a plexiform tumor composed of hypocellular and hypercellular areas with verocay bodies. The tumor cells showed strong positivity for S-100 protein, rendering a final diagnosis of plexiform schwannoma. The child has been free of recurrence in 12-month follow-up.

## INTRODUCTION

Plexiform schwannoma, a benign neoplasm, is an unusual morphologic variant of schwannoma. It is characterized by a plexiform or multinodular growth pattern.[1-3] It usually occurs in adolescents with involvement of superficial tissues of trunk, head, neck and upper extremities.[4] The most important differential diagnosis of plexiform schwannoma is plexiform neurofibroma which has a propensity for malignant transformation and strong association with neurofibromatosis.[3,5] We herein report a case of plexiform schwannoma found in an unusual location.

## CASE REPORT

A five-year-old female child presented with swelling in left lumbar region for eight months. It gradually increased in size and was associated with pain. There was no history of prior trauma or associated fever. No bladder or bowel complaints were noticed. Past medical and family histories were insignificant. On examination, a 10 cm x10 cm lobulated firm swelling was identified over the lower back. Skin overlying the swelling was unremarkable. No sensory or motor deficits were present. Systemic examination was unremarkable.

Magnetic resonance imaging (MRI) showed a multinodular plexiform tumor within the subcutaneous planes of lumbosacral region, extending from L1 to S1 vertebral levels. The tumor had similar intensity as muscle on T1-weighted images and was hyperintense on T2-weighted images (Fig. 1). No obvious intraspinal extension was noted. The radiologic features were suggestive of a subcutaneous multinodular tumor with possibility of plexiform neurofibroma or schwannoma.

**Figure:**
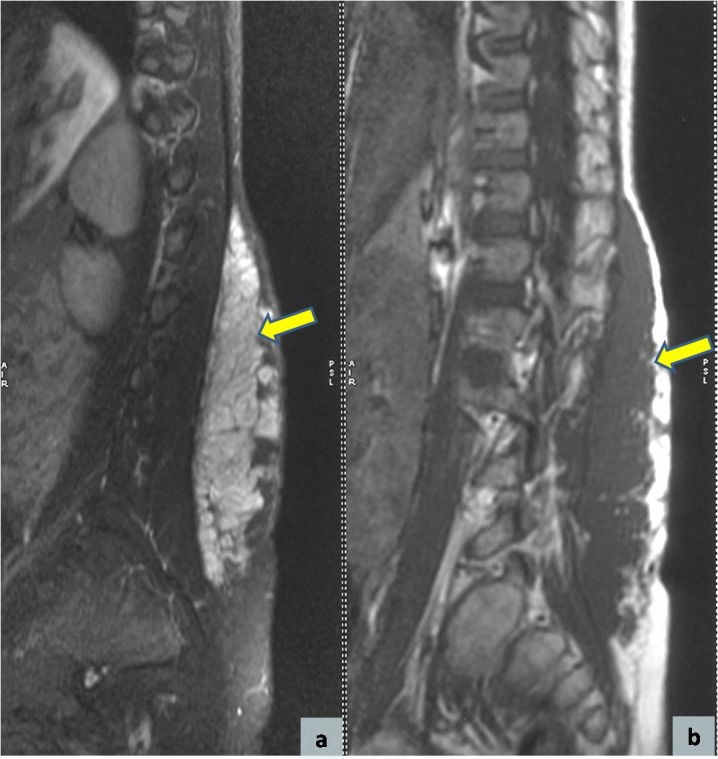
Figure 1: Magnetic resonance imaging (MRI) scans showing an elongated multinodular subcutaneous tumor (arrows), hyperintense on T2W (a) and isointense as muscle on T1W image (b).

Based on these findings, surgical excision was planned. Per-operatively, an infiltrating tumor was identified and excised. On cut section, all were grey-white, myxoid and nodular in appearance. Histopathological examination showed a multinodular tumor composed of spindle shaped cells arranged in interlacing fascicles (Fig. 2). There were few hypocellular areas with spindle cells present haphazardly in a loosely textured myxoid matrix. The spindle cells had elongated eosinophilic cytoplasm, wavy nuclei, fine chromatin and inconspicuous nucleoli. The cellular foci displayed Verocay bodies, formed by palisading of nuclei and separated by cell processes (Fig 2). There was no nuclear pleomorphism or any degenerative change. Mitotic activity was not increased and necrosis was not identified in multiple sections examined. On immunohistochemistry, tumor cells were strongly positive for S-100 protein (Fig. 2) and negative for smooth muscle actin. Hence a final pathologic diagnosis of plexiform schwannoma was made. Our patient is asymptomatic and showed no sign of recurrence on one year clinical follow up.

**Figure:**
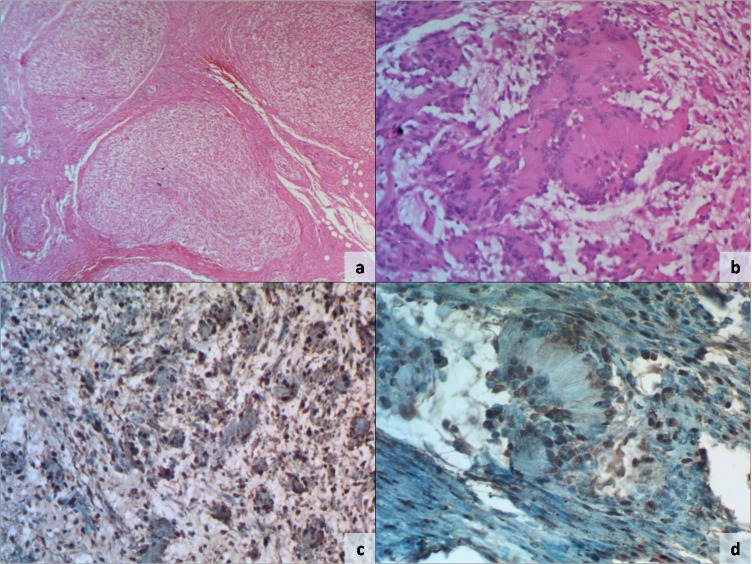
Figure 2: Histologic photomicrographs demonstrating a multinodular tumor (a, H and E x40) composed of spindle cells in a myxoid background with few Verocay bodies (b, H and E x200). Immunohistochemical stain for S-100 protein shows intense nuclear and cytoplasmic positivity in spindle cells (c, x100) as well as Verocay bodies (d, x200).

## DISCUSSION

Plexiform schwannoma is a rare, benign, peripheral nerve sheath tumor.[2] It is generally a sporadic, solitary, slow-growing nodule arising from the superficial (cutaneous or subcutaneous) tissues. It is usually reported in adolescents and young adults. The index case was five years of age. Plexiform schwannoma usually presents as an asymptomatic mass but occasionally it can be painful or tender as happened in the present case. The associations seen with this tumor, although weak, include neurofibromatosis-2 and schwannomatosis.[3,5] Schwannomatosis is a sporadic condition with two or more pathologically proven schwannomas.[6] Our patient had a solitary plexiform lesion.

The main differential diagnosis includes plexiform neurofibroma.[3] This differential is of utmost importance because of the fact that neurofibroma can undergo malignant transformation, which has not been the case with plexiform schwannoma.[3] The other differential is malignant peripheral nerve sheath tumor which requires wide excision.[7] In contrast, plexiform schwannoma can be treated by marginal excision.

On light microscopic examination, the plexiform or multinodular growth pattern of plexiform schwannoma mimics that of plexiform neurofibroma. However, the latter lacks the hypercellular Antoni A areas with presence of Verocay bodies and hypocellular myxoid Antoni B elements.[4,8] Neurofibroma demonstrates a weak and patchy immune-positivity with S-100 as compared to the diffuse and strong positivity of plexiform schwannoma.[8] Our case conformed to these features of plexiform schwannoma.

Since plexiform schwannoma is a benign tumor, complete surgical excision with free margins is the treatment of choice.[4] Our patient has been recurrence-free in the one year post-excision follow up.

In conclusion, plexiform schwannoma is a rare subtype of schwannoma that needs to be differentiated from plexiform neurofibroma and malignant peripheral nerve sheath tumor. Adequate sampling and careful histologic examination assists in an accurate diagnosis. This unusual tumor should be considered in differential diagnosis of superficial or subcutaneous plexiform soft tissue lesions.

## Footnotes

**Source of Support:** Nil

**Conflict of Interest:** None declared

